# Estimating the causal effect of embryo transfer day on clinical in vitro fertilization outcomes using propensity score matching

**DOI:** 10.1186/s12884-021-04013-y

**Published:** 2021-08-13

**Authors:** Han-Chih Hsieh, Chun-I Lee, En-Yu Lai, Jia-Ying Su, Yi-Ting Huang, Wei-Lin Zheng, Chien-Hong Chen, Chun-Chia Huang, Pin-Yao Lin, Maw-Sheng Lee, Mark Liu, Yen-Tsung Huang

**Affiliations:** 1 Institute of Statistical Science, Academia Sinica, No. 128 Academia Road, Taipei, 11529 Taiwan; 2 Institute of Epidemiology and Preventive Medicine, College of Public Health, National Taiwan University, Room 501, No. 17, Xu-Zhou Road, Taipei, 100 Taiwan; 3 Institute of Medicine, Chung Shan Medical University, No.110,Sec.1,Jianguo N.Rd., Taichung, 40201 Taiwan; 4 Department of Obstetrics and Gynecology, Chung Shan Medical University, No.110,Sec.1,Jianguo N.Rd., Taichung, 40201 Taiwan; 5 Division of Infertility, Lee Women’s Hospital, No. 30-6, Section 1, Changping Road, Taichung, 406 Taiwan; 6 Binflux Inc, 4F.-1, No. 9, Dehui St., Taipei, 104 Taiwan

**Keywords:** Blastocyst, Cleavage-stage transfer, In vitro fertilization, Embryo transfer, Indication bias

## Abstract

**Background:**

For women undergoing in vitro fertilization (IVF), the clinical benefit of embryo transfer at the blastocyst stage (Day 5) versus cleavage stage (Day 3) remains controversial. The purpose of this study is to compare the implantation rate, clinical pregnancy rate and odds of live birth of Day 3 and Day 5 embryo transfer, and more importantly, to address the issue that patients were chosen to receive either transfer protocol due to their underlying clinical characteristics, i.e., confounding by indication.

**Methods:**

We conducted a retrospective cohort study of 9,090 IVF cycles collected by Lee Women’s Hospital in Taichung, Taiwan from 1998 to 2014. We utilized the method of propensity score matching to mimic a randomized controlled trial (RCT) where each patient with Day 5 transfer was matched by another patient with Day 3 transfer with respect to other clinical characteristics. Implantation rate, clinical pregnancy rate, and odds of live birth were compared for women underwent Day 5 transfer and Day 3 transfer to estimate the causal effects. We further investigated the causal effects in subgroups by stratifying age and anti-Mullerian hormone (AMH).

**Results:**

Our analyses uncovered an evidence of a significant difference in implantation rate (*p*=0.04) favoring Day 5 transfer, and showed that Day 3 and Day 5 transfers made no difference in both odds of live birth (*p*=0.27) and clinical pregnancy rate (*p*=0.11). With the increase of gestational age, the trend toward non-significance of embryo transfer day in our result appeared to be consistent for subgroups stratified by age and AMH, while all analyses stratified by age and AMH were not statistically significant.

**Conclusions:**

We conclude that for women without strong indications for Day 3 or Day 5 transfer, there is a small significant difference in implantation rate in favor of Day 5 transfer. However, the two protocols have indistinguishable outcomes on odds of live birth and clinical pregnancy rate.

**Supplementary Information:**

The online version contains supplementary material available at (10.1186/s12884-021-04013-y).

## Background

There has been a long debate on whether the blastocyst stage (Day 5) embryo transfer should be routinely recommended to improve the implantation rate. [1–3] The introduction and improvements of advanced cell culture techniques have suggested blastocyst stage embryo transfer over cleavage stage (Day 3) embryo transfer for in vitro fertilization (IVF). Many published studies have suggested that Day 5 transfer provides higher implantation, pregnancy, and live birth rates [4–6]. However, others have argued that patients undergoing blastocyst culture are expected to have fewer transferable embryos available, and fewer embryos cryopreserved [7, 8]. Although several studies including randomized controlled trials (RCTs) have already been conducted, many of which were underpowered, leaving the margin of benefit between Day 3 and Day 5 transfers unclear.

Observational studies without randomization serve as an alternative study design to collect more participants to increase statistical power. However, the large sample size in observational studies pays the price of potentially introducing bias into the study. For example, Day 3 and Day 5 transfers were usually prescribed for patients with different clinical characteristics [9]. Without proper randomization, the difference in live birth rate or other associated outcomes is not necessarily attributable to the day of transfer. The phenomenon represents a typical confounding by indication [10]. The indication of receiving a certain treatment may affect the outcome of interest and thus exerts an undue influence on the association of the treatment and the outcome. Therefore, a powerful causal inference approach is imperative to determine the effectiveness of cleavage stage embryo transfer versus blastocyst stage embryo transfer.

While RCTs are considered as the gold standard approach for estimating the causal effects of treatments or exposures on outcomes, they are largely limited by ethical considerations and other practical limitations. More importantly, the practical constraints of RCTs make it difficult to distinguish between a null effect and a promising finding limited by the statistical power due to its small sample size. To integrate the advantages of RCT and observational study, here we adopt a propensity score matching approach. “Propensity score matching” section enables us to artificially construct an RCT from an observational study under certain assumptions [11]. The notion of propensity score was first introduced to be the probability of receiving a particular treatment conditional on observed baseline variables [12]. For the past few decades, different propensity score methods have been developed to remove the confounding effects under the potential outcome framework [13–15]. “Propensity score matching” section entails forming matched pairs of treated and untreated subjects that share similar value of propensity scores. Once the matched sample has been formed, the causal effect of the treatment on an outcome can be easily accessed by standard statistical procedures. To answer our scientific question by implementing this particular causal method, we had dichotomous embryo transfer day (Day 3 vs. Day 5) as treatment, implantation rate, clinical pregnancy rate, and odds of live birth as outcomes.

Our main purpose of this study was therefore to identify the causal effect of embryo transfer day on multiple major clinical outcomes in IVF. Additionally, we examine whether the causal effect of embryo transfer day on these outcomes can be modified by age and anti-Mullerian hormone (AMH) levels.

## Methods

### Patient selection

**Fig. 1:**
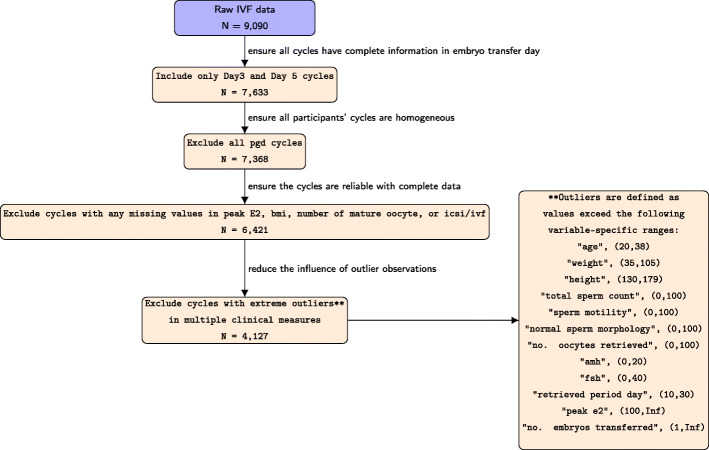
CONSORT diagram for data processing

A retrospective cohort analysis was conducted using a dataset collected from Lee Women’s Hospital in Taichung, Taiwan. This study was approved by the Institutional Review Board on Biomedical Science Research at Academia Sinica. The dataset we obtained consisted of 9,090 fresh cycles from 1998 to 2014. This is a protocol based upon the first fresh transfer after oocyte retrieval and cryopreservation is just considered between the covariates in order to describe better patients characteristics. The information recorded in this database for each cycle included patients’ age at cycle start, the date of oocyte retrieval, cycle status, cycle outcome, AMH, follicle stimulating hormone (FSH), estradiol (E2), along with other 64 variables. In order to explore the causal effects of embryo transfer day on odds of live birth and other associated outcomes, only patients with transfer day of Day 3 and Day 5 and without missing or incomplete outcome information were included. To minimize the potential bias caused by numerical outliers, cycles with outliers in certain variables, such as age, weight, height, total number of oocytes retrieved, total sperm count, sperm motility, normal sperm morphology, AMH, FSH, period day of retrieval, number of embryos transferred and peak E2 were excluded. Cycles with PGD/PGS (preimplantation genetic diagnosis/preimplantation genetic screening) cases were also excluded. Therefore, the final dataset contained 4,127 autologous IVF cycles, with known embryo transfer day, and confirmed information about live births, number of gestational sacs, and number of fetal heartbeats at 4 weeks of gestation. Details about the inclusion and exclusion criteria of the study were documented as a flowchart in Fig. 1. Controlled ovarian stimulation was achieved by a downregulation protocol in which patients were administered leuprolide acetate (Lupron, Takeda Chemical Industries, Ltd., Osaka, Japan) during the midluteal phase. Subsequently, patients received recombinant follicle stimulating hormone (Gonal-F; Serono, Bari, Italy) on day 3 for ovarian stimulation. The retrieved oocytes were cultured in Quinn’s Advantage Fertilization Medium (Sage Bio- Pharma, Inc., Trumbull, CT, USA) with 15% serum protein substitute (SPS, Sage BioPharma, Inc) at 37^∘^C with the gas mixture of 5% oxygen, 5% carbon dioxide and 90% of nitrogen. Following conventional insemination or intracytoplasmic sperm injection (ICSI), a fertilization medium (SAGE Biopharma, USA) with 15% serum protein substitute (SPS; SAGE Biopharma, USA) was used to further culture the embryos. 70 to 72 hours after insemination or ICSI, all cleaved embryos were group cultured in microdrops of a blastocyst medium (Sage BioPharma, Inc.) with 15% SPS. Further details about the clinical aspects of the IVF protocols or embryology for the study were documented in a previous paper [16].

### Statistical methods

#### Comparison of baseline characteristics and handling missing data

To observe the discrepancies of marginal characteristics of the clinical characteristics between patients undergoing Day 3 embryo transfer and Day 5 embryo transfer, a series of statistical tests were performed. We applied Welch’s t-tests for continuous variables, chi-square tests for binary variables, and Poisson regressions for count variables. The comparisons of baseline characteristics were conducted both before and after matching to evaluate the performance of our constructed propensity score. Because data were largely missing in peak E2, we utilized Generalized Association Plots (GAP) [17] to retrieve the important variables highly associated with peak E2. A single imputation for peak E2 was then performed using the number of embryos frozen, number of oocytes retrieved, number of follicles, and embryo transfer day. In order to avoid the confounding effect of prescribed dosage given by multiple physicians, we converted Gonal-F (follitropin alfa), Menotropin and recombinant luteinizing hormone (rLH) into dichotomous values, and combined Menotropin and rLH into a single variable (whether or not the patient was injected with either Menotropin or rLH throughout the cycle).

#### Propensity score matching

To estimate the causal effect of embryos transfer day on different clinical outcomes, we implemented the method of propensity score matching to mimic a randomized controlled trial. The propensity score of receiving Day 5 transfer was constructed by a logistic regression where the probability of receiving Day 5 embryo transfer was modeled through a logit link function. The formula and deviation of the propensity score are detailed in Supple-ment. Embryo transfer day was regressed on a series of observed baseline covariates to derive a propensity score for each IVF cycle. The baseline covariates selected in the propensity score model were based on the literature as well as their clinical implications [18]. The variables (*Xji*,*j*=1,...,*J*) contributed to the propensity score included: age (linear and quadratic terms), body mass index (BMI), AMH, tubal factor (divided into six categories: undecided, removed, patent, sticking, ligation and obstructed for both left and right side of Fallopian tubes), sperm motility, sperm count, normal sperm morphology, azoospermia, polycystic ovarian syndrome (PCOS), Gonal-F, menotropin, rLH, peak estradiol, day of trigger, number of follicles, number of oocytes retrieved, number of embryos transferred, number of embryos frozen, number of mature oocytes and ICSI vs. IVF. Furthermore, to adjust for the secular trend regarding changes or modifications of the protocols made by clinical professionals, the year of cycles performed was also included as an additional matching factor, i.e., we only paired cycles that the embryos were implanted in the same years. Matched sets of Day 3 and Day 5 transfer subjects were thus formed: for each cycle whose embryo transfer was Day 5, we selected another cycle with the nearest propensity score from those with an embryo transfer Day 3. The method of greedy matching was used to produce balanced matched samples without replacement [19]. Those without matched cycles from the other transfer protocol were excluded from the subsequent analyses.

#### Post-matching analyses

Using the matched dataset, the effect of embryo transfer day on odds of live birth was then estimated through logistic regression, adjusting for propensity score and the remaining covariates that appeared to be statistically different between Day 3 and Day 5 transfers after propensity score matching. On the other hand, the effect of embryo transfer day on the number of gestational sacs and the number of fetal heartbeats was then estimated by Poisson regression where the number of embryos transferred was treated as an offset. Similarly, the Poisson regression adjusted for propensity score and the remaining significant covariates to account for residual confounding.

#### Stratification by age and AMH

Additional analyses were conducted stratifying cycles by age and AMH. We stratified cycles based on the age categories recommended by SART (age <35, 35 ≤age<37, 37 ≤age≤38 years) and the quantiles of AMH (AMH =0, 0 ≤AMH≤1.39, 1.39 <AMH≤3.95, AMH >3.95 ng/ml). The method of propensity score matching, logistic regressions and Poisson regressions were applied repeatedly to each subgroup to identify the causal effects of embryo transfer day on associated IVF outcomes.

## Results

### Crude analyses

The clinical characteristics, live birth rate and other IVF associated outcomes among patients with Day 3 and Day 5 transfers before “Propensity score matching” section, were shown in Table 1. The crude live birth rate was slightly higher among those with Day 5 transfer (34.2% vs. 32.1%, *p* = 0.23); Day 5 transfers also showed advantage over Day 3 transfers on the average number of sacs (0.84 vs. 0.70, *p* <0.001) and number of fetal heartbeats (0.75 vs. 0.65, *p* = 0.001). The average age of patients in Day 3 was higher than that in Day 5 (32.89 vs. 31.61, *p*<0.001). For patients who had their embryos transferred on Day 3, the average AMH was much lower than that in group Day 5 (1.87 vs. 4.35, *p*<0.001), and the number of oocytes retrieved was fewer in those with Day 3 transfer (11.17 vs. 20.65, *p*<0.001). Other significantly different characteristics include BMI, azoospermia, PCOS, ICSI, Gonal-F, menotropin/rLH, peak E2, right duct, number of follicles, number of matured oocytes, and number of embryos frozen. As shown in Table 1, 16 out of 23 clinical characteristics were statistically significantly different between women receiving Day 3 transfer and those with Day 5, suggesting the two groups of patients had markedly distinct underlying features.

**Table 1:** Demographic characteristics before and after matching

	Before matching			After matching		
Variables	Day 3	Day 5		Day 3	Day 5	
	N=3172	N=955	*p*-val	N=577	N=577	*p*-val
Age (years)	32.89 ±3.57	31.61 ±3.60	<0.001	31.77 ±3.63	31.76 ±3.62	0.98
BMI (kg/m^2^)	21.78 ±3.14	22.09 ±3.53	0.02	22.20 ±3.27	22.11 ±3.51	0.64
Azoospermia (%)	3.6	1.9	0.01	2.8	2.3	0.71
PCOS (%)	0.7	2.8	<0.001	1.7	2.1	0.83
ICSI/IVF	1	2	<0.001	2	2	0.05
AMH (ng/ml)	1.87 ±2.51	4.35 ±4.04	<0.001	3.73 ±3.34	3.77 ±3.63	0.83
Gonal-F (%)	99.3	97.0	<0.001	97.2	97.6	0.85
Menotropin/rLH (%)	57.7	27.5	<0.001	27.0	29.6	0.36
Peak E2 (pg/ml)	1492.3 ±1059.9	3216.3 ±1685.0	<0.001	2488.8 ±1559.9	2512.9 ±1186.7	0.77
Total sperm count (/ml)	34.46 ±30.70	32.69 ±31.19	0.12	29.74 ±30.42	32.09 ±31.04	0.19
Sperm motility (%)	46.5	44.4	0.10	42.2	44.3	0.31
Normal sperm morphology (%)	7.2	6.6	0.05	6.0	6.2	0.64
Left duct	3	3	0.05	3	3	0.84
Right duct	3	3	0.007	3	3	0.43
No. follicles	11.96 ±9.47	21.89 ±9.47	<0.001	18.62 ±17.30	18.39 ±7.34	0.35
Day of trigger	12.14	11.97	0.21	12.02	12.07	0.79
No. oocytes retrieved	11.17 ±6.73	20.42 ±9.40	<0.001	17.34 ±7.77	17.13 ±7.37	0.39
No. mature oocytes	8.97 ±5.51	17.08 ±8.09	<0.001	14.36 ±6.36	14.14 ±6.19	0.32
No. embryos transferred	3.69 ±1.63	3.62 ±1.50	0.29	3.84 ±1.30	3.65 ±1.57	0.10
No. embryos frozen	0.51 ±1.43	2.24 ±3.37	<0.001	1.50 ±2.34	1.64 ±2.77	0.046
No. gestational sacs	0.70 ±1.04	0.84 ±1.16	<0.001	0.78 ±1.07	0.85 ±1.19	0.20
No. fetal heartbeats	0.65 ±1.07	0.75 ±1.14	0.001	0.72 ±1.04	0.76 ±1.16	0.37
Live Birth (%)	32.1	34.2	0.23	36.6	33.6	0.32

For continuous and count variables, means ± SDs were presented.

*BMI* body mass index;

*PCOS* Polycystic ovary syndrome;

*ICSI/IVF* 0 (cycle with only IVF embryos), 1 (cycle with only ICSI embryos), 2 (cycle mixed with both IVF and ICSI embroys);

*AMH* Anti-Mullerian hormone;

*Gonal-F* follitropin alfa;

*E2* Estradiol;

*Left duct/ right duct* 1 (undecided), 2 (remove), 3 (patent), 4 (sticking), 5 (ligation), 6 (obstructed);

*Live birth* 0 (No live birth delivery), 1 (one or more than 1 live birth delivery)

### Post matching analyses

**Fig. 2:**
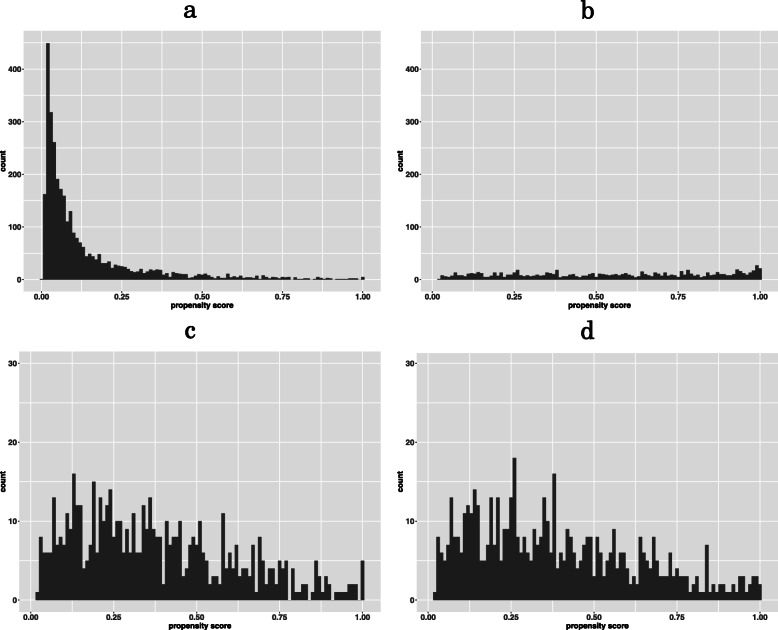
Propensity score distribution: a. Day 3 before matching; b. Day 5 before matching; c. Day 3 after matching; d. Day 5 after matching

After we matched our data using propensity score, only 577 patients were matched in each group, indicating that many patients had strong clinical indications for either Day 3 or Day 5 transfer and thus may not be matched with those receiving the other transfer protocol. Only one variable, the number of embryos frozen, out of 23 variables was still significantly different across the two groups after matching (Table 1). The distributions of the propensity scores across two groups before and after matching are shown in Fig. 2.

A logistic regression was used to access the causal effect of embryo transfer day on odds of live birth, adjusting for the number of embryos frozen (Table 2). The analyses showed that embryo transfer day was not associated with odds of live birth (odds ratio 0.87 [95% confidence interval: 0.68, 1.11], *p* = 0.27). Our Poisson regression analyses also showed that embryo transfer day was not associated with clinical pregnancy rate (number of fetal heartbeats per embryo transferred; *p* = 0.11). Nevertheless, our analysis revealed a risk ratio (RR) of 1.14 in favor of Day 5 transfer in implantation rate (number of gestational sacs per embryos transferred; RR 1.14 [95% confidence interval: 1.0004, 1.30], *p*=0.04).

**Table 2:** Regression model estimating associated outcomes after matching

Variables	Implantation rate		Clinical Pregnancy rate		Odds of live birth	
	Estimate (95% CI)	*p*-val	Estimate (95% CI)	*p*-val	OR (95% CI)	*p*-val
Embryo transfer day	0.13 (0.004, 0.26)	0.04	0.11 (-0.02, 0.24)	0.11	0.87 (0.68, 1.11)	0.27
Propensity score	0.28 (-0.01, 0.56)	0.06	0.31 (0.01, 0.61)	0.04	1.08 (0.63, 1.85)	0.78
No. embryos frozen	0.02 (-0.001, 0.048)	0.06	0.02 (-0.002, 0.05)	0.07	1.05 (1.00, 1.10)	0.08

Implantation rate: number of gestational sacs/ number of embryos transferred.

Clinical Pregnancy rate: number of fetal heartbeats present/ number of embryos transferred.

### Stratified analyses

We further investigated the association between day of embryo transfer and IVF outcomes among subgroups of age and AMH, again using propensity score matching. Unlike our main analyses in Table 3, where blastocyst stage transfer is significantly associated with implantation rate, the “Stratified analyses” section by age (Table 3) and AMH (Table 4) showed that there was no evidence for association of embryo transfer day and IVF outcomes across all subgroups.

**Table 3:** Regression model estimating associated outcomes after matching stratified by age

		Variables	Implantation rate		Clinical Pregnancy rate		Odds of live birth	
			Estimate (95% CI)	*p*-val	Estimate (95% CI)	*p*-val	OR (95% CI)	*p*-val
Age <35	N= 858	Embryo transfer day	0.09 (-0.05, 0.23)	0.23	0.05 (-0.09, 0.20)	0.48	0.83 (0.62, 1.09)	0.18
		Propensity score	0.04 (-0.26, 0.35)	0.78	0.13 (-0.19, 0.45)	0.43	0.97 (0.53, 1.74)	0.91
35 ≤Age <37	N= 146	Embryo transfer day	0.17 (-0.23, 0.58)	0.40	0.21 (-0.23, 0.65)	0.35	1.13 (0.55, 2.32)	0.73
		Propensity score	0.57 (-0.17, 1.31)	0.13	0.46 (-0.34, 1.27)	0.26	3.67 (0.99, 13.54)	0.05
37 ≤Age≤38	N= 100	Embryo transfer day	0.35 (-0.17, 0.86)	0.19	0.26 (-0.27, 0.80)	0.33	1.28 (0.52, 3.12)	0.59
		Propensity score	1.11 (0.10, 2.12)	0.03	1.20 (0.16, 2.24)	0.02	4.64 (0.64, 33.43)	0.13
		No. embryos frozen	-0.11 (-0.30, 0.08)	0.24	-0.12 (-0.33, 0.08)	0.23	0.88 (0.64, 1.22)	0.45

Implantation rate: number of gestational sacs/ number of embryos transferred

Clinical Pregnancy rate: number of fetal heartbeats present/ number of embryos transferred

**Table 4:** Regression model estimating associated outcomes after matching stratified by AMH

		Variables	Implantation rate		Clinical Pregnancy rate		Odds of live birth	
			Estimate (95% CI)	*p*-val	Estimate (95% CI)	*p*-val	OR (95% CI)	*p*-val
AMH=0	N= 320	Embryo transfer day	0.15 (-0.08, 0.38)	0.21	0.03 (-0.22, 0.28)	0.80	0.94 (0.58, 1.50)	0.78
		Propensity score	-0.03 (-0.52, 0.47)	0.91	0.04 (-0.48, 0.55)	0.88	1.57 (0.59, 4.17)	0.37
		No. embryos frozen	0.01 (-0.03, 0.06)	0.53	0.02 (-0.03, 0.06)	0.48	1.02 (0.93, 1.12)	0.65
		ICSI/IVF	-0.002 (-0.15, 0.15)	0.98	0.05 (-0.11, 0.21)	0.56	0.95 (0.70, 1.29)	0.73
0 ≤AMH≤1.39	lN= 16	Embryo transfer day	-0.30 (-1.57, 0.98)	0.65	-1.71 (-3.83, 0.42)	0.12	0.21 (0.02, 2.96)	0.25
		Propensity score	-0.69 (-5.20, 3.80)	0.76	-0.03 (-5.17, 5.12)	0.99	22.34 (0.006, 91416.5)	0.46
1.39 <AMH≤3.95	N=236	Embryo transfer day	0.15 (-0.15, 0.43)	0.34	0.20 (-0.11, 0.51)	0.20	1.03 (0.61, 1.76)	0.90
		Propensity score	0.16 (-0.47, 0.78)	0.63	0.27 (-0.38, 0.93)	0.41	1.68 (0.54, 5.21)	0.37
AMH >3.95	N= 482	Embryo transfer day	0.03 (-0.17, 0.23)	0.79	0.002 (-0.21, 0.20)	0.99	0.90 (0.62, 1.30)	0.57
		Propensity score	0.42 (-0.04, 0.88)	0.07	0.40 (-0.08, 0.88)	0.10	0.98 (0.41, 2.36)	0.97

Implantation rate: number of gestational sacs/ number of embryos transferred

Clinical Pregnancy rate: number of fetal heartbeats present/ number of embryos transferred

## Discussion

The principal finding of this study was that, comparing the women who shared similar baseline characteristics, there was evidence of a small but significant difference in implantation rate favoring Day 5 embryo transfer and no difference in the probability of live birth and clinical pregnancy.

The outcome of implantation, which is defined as the number of gestational sacs per embryo transferred, is often considered as the earliest primary endpoint in the process of IVF, and may affect the success of delivery. Our result showed that the blastocyst stage group had a higher rate of implantation, a relative risk of 1.14, than the group of cleavage stage transfer. This finding is in support of the previous studies that reported Day 5 transfers have purported advantages based on its morphological features [20, 21]. As the consequence of self selection, only the most viable embryos are expected to develop into the 64-cell blastocysts in the in vitro environment, eliminating those who had chromosomal abnormalities at an early age [22, 23]. Meanwhile, studies have also shown that the predictive morphological criteria of the embryos expected to be transferred on Day 3 are limited [24–26]. In addition, it is recognized that premature exposure of early stage embryos to the uterine environment may induce homeostatic stress, reducing the potential of successful implantation [27]. This finding is also consistent with numerous trials reporting higher implantation rates for blastocyst transfer [28–30].

Although blastocyst transfer is an effective procedure of increasing implantation rate, our result revealed that the transfer of embryos on Day 5 rather than on Day 3 did not change the overall probabilities of pregnancy and live birth. One of the potential cause for this phenomenon may be the existence of the post-treatment confounding similar to that of the lack of blinding effect in an RCT [31]. Blinding refers to maintaining unawareness of the assigned interventions among trial participants, healthcare providers, and assessors. However, even the goal of our method is to mimic an RCT in the context of an observational study, none of the people mentioned above in our study were blinded, which may leave room for potential bias due to residual confounding. For example, the clinicians may reflect their attitudes, paying extra attention or making differential decisions on the management, based on the allocation. Likewise, the patients may be more likely to comply with the clinical care after knowing they are about to or already had been assigned to Day 3 embryo transfer. Our propensity score was constructed based on the variables before implantation. However, numerous post-implantation factors may still affect the live birth rate. Even though blinding may reduced bias, the length of culture and the different procedures required based on the day of embryo transfer make it impossible to blind which group the patients were in for the technicians and clinicians.

Recent paper has revealed that serum AMH level is positively correlated with ovarian responsiveness, embryo developmental competence, and cumulative live birth rate [32]. Therefore, we further explored the effect of embryo transfer day on live birth and other associated outcomes by categorizing patients’ age and AMH. Interestingly, we were able to identify a trend that is consistent with our main finding: an increase in *p* values of embryo transfer day on IVF outcomes as the gestational age increases from 14 days (implantation rate) to week 4 (clinical pregnancy). For instance, the group where patients’ age were under 35, the *p* values of embryo transfer day increased from 0.23 to 0.48 as the outcome progressed from implantation rate to clinical pregnancy rate; as well as the group of patients aged between 37 and 38, the *p* values increased from 0.19 to 0.33. Although this trend was preserved in both our main result and stratified results, there was no evidence of significant difference in the later.

A recently published systematic review has performed meta-analysis on 27 RCTs to determine whether blastocyst transfers improve live birth and other associated IVF outcomes compared with cleavage stage transfers [33]. The study has revealed very similar results on clinical pregnancy rates with ours, both did not find difference between Day 3 transfer and Day 5 transfer. Yet, the review did report an evidence of a significant difference in live birth rate between the two groups, favoring Day 5 transfer. It is important to note that, although there is discrepancy between the two results on live birth, the intervention between the two studies are not the same. The previous study compared Day 2 and Day 3 transfers with Day 5 and Day 6 transfers, whereas our study only compared Day 3 transfers with Day 5 transfers.

Our results showing no evidence of increasing odds of live birth in Day 5 embryo transfer is consistent with a recent cohort conducted in the UK [34]. Although our studies share similar study design, the patient population between the two studies are different. A growing body of existing literature have indicated the racial and ethnic disparities that may appear in assisted reproductive technology outcomes. Current evidence have demonstrated the predisposition of Asian women having worse IVF outcomes, which may be resulting from fundamental biological or genetic differences [35, 36]. For example, studies have shown the distribution of FSH receptor allelic variants varies among Asians and Caucasians [37]. Alternatively, behavioral and environmental differences may have also caused the decreased pregnancy rate in Asian women [38, 39]. To our knowledge, our study is the biggest cohort in Asian population in assessing the differences between blastocyst stage transfer and cleavage stage transfer.

The goal of propensity score matching is to mimic an RCT from an observational study. The validity of this approach depends on a few assumptions. First, the propensity of receiving Day 5 transfer was properly modeled. The tendency of physician’s preference in prescribing a certain transfer protocol is unknown. On the other hand, to reduce the potential multi-dimensional confounding effect into a one-dimensional propensity score (PS) itself is a strong assumption. The assumption states that the unknown tendency is fully captured by the logistic model (model (1) in Supplement). It requires that all relevant variables are included and the feature of the variables (linear, quadratic, categorical, cross-product between variables, etc.) is correctly specified. However, limitations may arise in practice when we excluded certain highly correlated variables, such as endometriosis and ovarian cancer, in order to adjust the confounding effects and produce a homogeneous dataset. This may threaten the statistical generalizability of this study. Second, the tolerance of imperfect matching did not result in residual confounding. Numerically, we may not find a perfect match with an identical PS and systematic imperfect matches may lead to confounding. We address this issue by additionally adjust for propensity score in the “Post-matching analyses” section.

As an RCT require a carefully selected participants to ensure internal validity, its generalizability to population that does not satisfy its inclusion criteria is of concern. A similar issue applies in the PS matching approach. As shown in Fig. 2., the propensity scores between Day 3 and 5 transfers were quite different before matching, indicating that there are patients that were very likely to receive Day 3 transfer and unlikely to receive Day 5 transfer and vice versa. After matching, we only preserve those whose tendency of receiving either transfer is not that high. This phenomenon reflects the clinical equipoise, a common basis for clinical trials. Therefore, focusing on the patients who are equivocal for their tendency of receiving either transfer is a consequence of mimicking an RCT. However, what inherits from the equipoise principle is its difficulty in generalize the result to the patients not included in our matched data. For example, the null effect of Day 3 and Day 5 transfers may not be applicable to patients who have strong clinical indications of receiving Day 3 or Day 5 transfer. We stress that our matched analyses results need to be cautiously interpreted.

## Conclusion

In “Conclusion” section, this study provides evidence that there is difference in implantation rate favoring Day 5 transfer compared to Day 3 transfer. However, considering later IVF outcomes, such as clinical pregnancy and live birth delivery, we suggest that advising women who share equivocal baseline characteristic at consultation to initiate an IVF cycle leading to a Day 5 transfer does not appear to increase the ongoing clinical pregnancy rate and odds of live birth compared with initiating an IVF cycle leading to Day 3 transfer.

## Supplementary Information

**Additional file 1** Deviation and formula for propensity score.

## Data Availability

The datasets used and/or analysed during the current study are available from the corresponding author on reasonable request.

## Abbreviations

Day 3 embryo transfer: Cleavage stage embryo transferred on day 3
Day 5 embryo transfer: Blastocyst stage embryo transferred on day 5
RCT: Randomized controlled trial
AMH: Anti-Mullerian hormone
BMI: Body mass index
IVF: In vitro fertilization
FSH: Follicle stimulating hormone
E2: Estradiol
PGD/PGS: Preimplantation genetic diagnosis/preimplantation genetic screening
GAP: Generalized Association Plots
Gonal-F: Follitropin alfa
rLH: Recombinant luteinizing hormone
PCOS: Polycystic ovarian syndrome
PS: Propensity score
CI: Confidence interval
OR: Odds ratio
