# Supplementary material for: Estimating the causal effect of embryo transfer day on clinical in vitro fertilization outcomes using propensity score matching
Source: BMC Pregnancy Childbirth. 2021 Aug 13;21:554. doi: 10.1186/s12884-021-04013-y (PMC8364091; doi:10.1186/s12884-021-04013-y)
Supplement: Supplementary file 1 — Additional file 1 Deviation and formula for propensity score. [file 12884_2021_4013_MOESM1_ESM.pdf]

Supplementary Materials for "Estimating the Causal Effect of Embryo Transfer Day on Clinical In Vitro Fertilization Outcomes using Propensity Score Matching"

by

HC Hsieh*, CI Lee*, EY Lai, JY Su, YT Huang, WL Zheng, CH Chen, CC Huang, PY Lin, MS Lee, M Liu, YT Huang

0.1 Formula and Derivation of Propensity Score

We formed the following logistic model:

$$\log \frac{P(Y_i = 1 \mid X_{1i}, ..., XJi)}{1 - P(Y_i = 1 \mid X_{1i}, ..., XJi)} = \beta_0 + \sum_{j=1}^{J} \beta_j X_{ji}, \tag{1}$$

where Y_i is subject i receives Day 5 transfer and 0 if receiving Day 3 transfer, and X_{ji} is the covariate j for subject i.

The propensity score (PS) of subject i is constructed as:

$$PS_i = \hat{P}(Y_i = 1|X_{1i}, ..., XJi) = \frac{\exp(\hat{\beta}_0 + \sum_{j=1}^{J} \hat{\beta}_j X_{ji})}{1 + \exp(\hat{\beta}_0 + \sum_{j=1}^{J} \hat{\beta}_j X_{ji})}, \tag{2}$$

where $(\hat{\beta}_0, \hat{\beta}_1, ..., \hat{\beta}_J)$ are the estimates of $(\beta_0, \beta_1, ..., \beta_J)$ obtained from fitting the above regression model.
